# Prediction of live birth in vitrified-warmed 1PN-derived blastocyst transfer: Overall quality grade, ICM, TE, and expansion degree

**DOI:** 10.3389/fphys.2022.964360

**Published:** 2022-11-10

**Authors:** Tiantian Wang, Jiqiang Si, Bian Wang, Mingru Yin, Weina Yu, Wei Jin, Qifeng Lyu, Hui Long

**Affiliations:** Department of Assisted Reproduction, Shanghai Ninth People’s Hospital, Shanghai Jiao Tong University School of Medicine, Shanghai, China

**Keywords:** monopronuclear, morphology grading, pregnancy, live birth, prediction

## Abstract

**Background:** Numerous studies have reported that transfer of blastocysts derived from monopronuclear (1PN) zygotes achieved live births. However, the potential value of morphology grading for the prediction of 1PN blastocyst viability is unclear, and the blastocyst selection criterion for successful pregnancy has not been set up yet. The aim of this study is to assess the ability of the blastocyst morphology grading system based on three parameters, namely, inner cell mass (ICM), trophectoderm (TE), and expansion degree and to predict outcomes of a cycle with single 1PN blastocyst transfer.

**Methods:** A total of 266 vitrified-warmed 1PN-derived blastocyst transfer cycles for IVF treatment at Shanghai Ninth People’s Hospital between 2007 and 2020 were included. The study was performed on single blastocyst transfers. Electronic records of patients were retrospectively analyzed. In the current study, the blastocysts were classified into three groups: “good,” 3-6AA, 3-6AB, 3-6BA; “medium,” 3-6BB, 3-6AC, 3-6CA; and “poor,” 3-6BC, 3-6CB, 3-6CC. The basal characteristics, embryo grading, and clinical outcomes were compared between the three groups. The association of morphology parameters with pregnancies and live births was analyzed. Logistic regression was adopted to set up a prediction model of live births.

**Results:** Transfer of the good-quality blastocysts achieved significant higher pregnancies (biochemical pregnancy: 59%; clinical pregnancy: 56.4%, and live birth 48.7%) than those in the group of the medium (biochemical pregnancy: 59%; clinical pregnancy: 49.6%; live birth: 40.4%) or poor-quality (biochemical pregnancy: 38.4%; clinical pregnancy: 34.9%; live birth: 26.7%) blastocysts (*p* < 0.05). There was a significant association between ICM and live birth. A prediction model of live births involving ICM, TE, and expansion degree was set up.

**Conclusion:** In 1PN transfer cycles, a higher overall blastocyst quality is shown to correlate most strongly with optimal pregnancy and live birth outcomes. The selection of high-quality blastocysts for transfer should consider the ICM score first. The prediction model of live births based on ICM, TE, and expansion degree may help predict successful pregnancy in 1PN single-blastocyst transfer cycles.

## Introduction

In the field of *in vitro* fertilization–embryo transfer (IVF-ET), transfer of embryos originating from zygotes with normal chromosomal constitution is required for the satisfying clinical outcomes. Generally, the presence of two pronuclear (2PN) and bodies is the symbol of normal fertilization, indicating the normal chromosomal constitution, while the presence of non-pronuclear (0PN) or monopronuclear (1PN) is typically the abnormal fertilization. Although the embryos originated from normal fertilization zygotes were optimally selected for transfer, those 1PN-derived blastocysts were also the choice due to the incidence of diploid rates, especially in cases where no normal fertilization embryos were originated (Staessen and Van Steirteghem, 1997; Bradley et al., 2017; Capalbo et al., 2017).

Many studies reported that transferring 1PN-derived blastocysts also obtained the similar outcomes as the transfer of 2PN-derived blastocysts, including pregnancies and live births (Bradley et al., 2017; Si et al., 2019; Chen et al., 2020; Li et al., 2020; Li et al., 2021). If we discarded those embryos, some patients, especially older patients or patients with decreased ovarian reserve, will miss an opportunity for pregnancy. In clinical practice, some intervention, such as blastocyst culture for transfer, actually improved the potential of successful pregnancy after the transfer of the 1PN-derived embryos (Bradley et al., 2017; Si et al., 2019). To avoid the risk of adverse developmental outcomes of aneuploidy embryos, some genetic techniques, such as preimplantational genetic screening (PGS) with comprehensive chromosome screening (CCS) and time-lapse embryo monitoring, were applied to assess the implantation potential of 1PN-derived embryos (Mateo et al., 2017; Destouni et al., 2018). However, as a non-invasive, convenience, and economic method, the traditional morphology evaluation was still irreplaceable in clinical practice. So far, the accurate prediction of the developmental potential of the 1PN-derived blastocysts based on morphology is still lacking.

Embryo quality is considered a major predictor of implantation and pregnancy (Kemper et al., 2021; Anagnostopoulou et al., 2022). The blastocyst grade system based on blastocyst expansion, inner cell mass (ICM), and trophectoderm (TE) development provides a powerful prediction between the morphology quantitative measurement and pregnancy and live birth in normal fertilization embryo transfer (Gardner and Schoolcraft, 1999; Firmin and Maitre, 2021). Accumulating evidence proved the relationship of pregnancy and live birth with the composite scores of the different morphology parameters in multiple or single 2PN-derived blastocyst transfer (Guo et al., 2020; Maheshwari et al., 2022). Also, their correlations and the potential of each morphology parameter at the blastocyst stage in live birth prediction were also explored in 2PN-derived blastocyst transfer (Ahlstrom et al., 2016; Girard et al., 2018). Nevertheless, the values of the ICM, TE, and expansion degree in prediction of clinical outcomes have not been reported in 1PN-derived single blastocyst transfer yet.

In the current study, we retrospectively analyzed the clinical outcomes of 1PN-derived blastocyst transfer in frozen–thawed IVF cycles from 266 patients between April 2007 and May 2020 in Ninth People’s Hospital affiliated to Shanghai Jiao Tong University. First, the study aimed to investigate the relationship between individual morphology parameters and biochemical pregnancy, clinical pregnancy, and live birth using data on 1PN-derived single blastocyst transfers. Second, we developed a multiple logistic regression model for the prediction of the probability of live birth after 1PN-derived single blastocyst transfers based on blastocyst morphology grading for clinical practices.

## Materials and methods

### Patients and cycles

The present retrospective study was conducted at the Department of Assisted Reproduction of Ninth People´s Hospital affiliated to Shanghai Jiao Tong University School of Medicine involving transfer of 266 vitrified-warmed 1PN-derived blastocysts in IVF cycles during April 2007–May 2020. The study was performed on single blastocyst transfers. The patients lost to follow-up were excluded.

### Ethics statement

Approval for human retrospective analysis was obtained from the Institutional Ethics Committee of Shanghai Ninth People’s Hospital. All participants provided informed consent after counseling for infertility treatments and routine IVF procedures.

### Laboratory protocols

Oocytes were retrieved approximately 36 h after hCG administration. All the aspirated oocytes were transferred to the G-IVF PLUS culture media (Vitrolife, Sweden) and fertilized with the use of conventional insemination (IVF). In case of IVF, oocytes were inseminated with approximately 50,000 progressively motile spermatozoa/ml which were harvested with the use of density gradient and swim-up methods in the insemination dish. Zygotes were transferred to the dish containing preequilibrated G-1™ cleavage culture media (Vitrolife, Sweden) 16–18 h after insemination. Fertilization was assessed according to the presence of the pronuclear. Embryos obtained from 1PN zygotes were downgraded on day 3, and all were placed in the G-2™ blastocyst culture media (Vitrolife, Sweden) until the blastocyst stage was reached. Then, the blastocysts were vitrified and thawed in FET cycles. The vitrification procedure was conducted using the Cryotop carrier system (KITAZATO Biopharma Co.). The cryoprotectant solution (KITAZATO^@^ Vitrification Kit, Japan) consisted of 15% ethylene glycol, 15% dimethyl sulfoxide, and 0.5 M sucrose. We usually used laser objective and positioned the laser pointer on the zona pellucida at the opposite side of the ICM and drilled the zona pellucida through two to three laser pulses. The vitrification procedure collapsed the blastocysts within 30 min to prevent their re-expansion. When thawing blastocysts, the cryoprotectant dilutions (KITAZATO^@^ Vitrification Kit, Japan) were sequential: 1, 0.5, and 0 M sucrose solutions. All the steps were carried out at room temperature except the first warming step (37°C) (Shen et al., 2020). The blastocysts were usually thawed at approximately 9:30 a.m. and transplanted at 2:00 p.m.

On days 5, 6, and 7 of *in vitro* cultivation, the blastocysts were evaluated morphologically according to Gardner and Schoolcraft´s classification (Gardner and Schoolcraft, 1999). In our center, blastocyst grading was performed by experienced embryologists, which lowered embryologist variation. The blastocysts were assigned 3, 4, 5, and 6 numeric score based on the degree of expansion and hatching status. Stage 3 blastocysts: full blastocysts; the embryo was completely filled with blastocoel; Stage 4 blastocysts: expanded blastocysts; the blastocoel was larger and the zona was thinner; Stage 5 blastocysts: hatching blastocysts; the TE began to herniate through the zona; Stage 6 blastocysts: hatched blastocysts; the blastocysts completely escaped from the zona. In our center, the blastocysts graded three to six were used for FET. Then, the blastocyst morphology was assessed according to inner cell mass and TE. The ICM grade was evaluated as follows: A, tightly packed with many cells; B, loosely grouped with several cells; and C, few cells. The TE grade was evaluated as follows: A, formed a cohesive epithelium with many cells; B, formed a loose epithelium with few cells; and C, few large cells (Schoolcraft et al., 1999; Zhao et al., 2018).

In this study, the blastocysts were classified into three groups: “good,” 3-6AA, 3-6AB, 3-6BA; “media,” 3-6BB, 3-6AC, 3-6CA; and “poor,” 3-6BC, 3-6CB, 3-6CC (Capalbo et al., 2014; Irani et al., 2017; Zhao et al., 2018). All embryos were cultured in a benchtop incubator (ASTEC) at 37°C with 5% O_2_ and 6% CO_2_ concentration. The embryo check-out time was usually between 8 a.m. and 10 a.m. on days 5, 6, and 7. Sometimes, the observation time of the blastocysts on day 5 extends till afternoon.

### Embryo transfer and luteal support

In this study, no 1PN-derived blastocysts were transferred in fresh cycles because in our center a freeze-all strategy was adopted. FET was performed *via* a natural cycle or an artificial cycle according to the individual condition of the patient. Women with menstrual regularity underwent a natural cycle in which human chorionic gonadotropin (hCG, 5000IU; Lizhu Pharmaceutical Trading Co., Shanghai, China) was used as a trigger. Luteal support included Femoston tablets (4 mg/d, Abbott Healthcare Products B.V.) and soft vaginal progesterone capsules (0.4 g/d, Utrogestan, Laboratories Besins Iscovesco, France). Women with menstrual irregularity or abnormal vaginal bleeding history underwent an artificial cycle in which from the third day onward, oral 17ß-estradiol (Fematone 2 mg, three times daily) was commenced for 14 days. Luteal supplement was described as mentioned previously.

### Clinical outcomes

Biochemical pregnancy was defined as hCG-positive cycles. Clinical pregnancy was defined as the presence of a gestational sac with cardiac pulse on ultrasound examination, and the clinical pregnancy rate of analyzed cycles was calculated as the intrauterine and ectopic pregnancies divided by the number of embryos transferred cycles.

### Statistical analysis

All data statistical analyses were performed using SPSS software. All continuous data were tested for normality. Those normally distributed data were presented as mean ± standard deviation and were analyzed using Student´s *t*-test. Those non-normally distributed data were presented as the mean (interquartile range) and were subjected to the Mann–Whitney *U*-test. Categorical variables were expressed as percentages and were analyzed using the chi-squared test or Fisher´s exact test. The relationship between parameters and outcomes were analyzed *via* a simple logistic regression analysis. A multiple logistic regression model of the live birth rate was conducted using the three parameters: expansion, ICM grade, and TE grade entering into the model. Based on the model, the predicted probabilities of live birth accompanied with 95% Wald confidence limits were reported. A value of *p* < 0.05 was referred to as a statistical significance.

## Results

### Association of blastocyst grades with clinical outcomes of embryos originated from monopronuclear zygotes

A total of 266 frozen–thawed blastocysts originated from monopronuclear zygotes in IVF cycles were included in the current study. These blastocysts were divided into three groups according to morphological grades (good, medium, and poor) as showed in Table 1. There was no significant difference in patient age, BMI, years of infertility, type of infertility, and endometrial thickness between the three groups (Table 2).

**TABLE 1:** Number of transferred embryos in quality classifications.

Good (*n* = 39)	Medium (*n* = 141)	Poor (*n* = 86)			
Grade	*n*	Grade	*n*	Grade	*n*
3AB	0	3BB	1	4BC	48
4AB	19	4BB	117	5BC	2
5AB	2	5BB	9	6BC	0
6AB	1	6BB	11	4CB	20
4BA	8	3AC	0	5CB	6
5BA	0	4AC	3	6CB	4
6BA	0	5AC	0	3BC	2
3BA	0	6AC	0	3CB	0
3AA	0	3CA	0	3CC	2
4AA	9	4CA	0	4CC	2
5AA	0	5CA	0	5CC	0
6AA	0	6CA	0	6CC	0

The morphological characteristics were described by the ICM grade, TE grade, and the degree of blastocyst expansion; TE, trophectoderm; ICM, inner cell mass.

**TABLE 2:** Patient characteristics of embryos with different morphological grades between 2007 and 2020.

Patient characteristic	Good	Medium	Poor	*P*1	*P*2	*P*3
No. of transfers	39	141	86			
Age (years)	37.9 ± 4.99	36.82 ± 4.98	37.48 ± 4.67	0.232	0.649	0.322
BMI (kg/m^2^)	20.75 ± 2.08	21.58 ± 3.58	22.00 ± 3.75	0.165	0.053	0.406
Years of infertility	3.67 ± 3.04	3.51 ± 2.71	3.60 ± 2.85	0.757	0.912	0.804
Type of infertility, *n*						
Primary infertility	20	68	38	0.736	0.461	0.554
Secondary infertility	19	73	48			
Endometrial thickness	10.17 ± 1.77	10.22 ± 2.59	10.52 ± 2.54	0.908	0.437	0.396

Data were presented as mean ± SD or mean. *P*1, *p*-value between good and medium; *P*2, *p*-value between good and poor; *P*3, *p*-value between medium and poor; BMI, body mass index; SD: standard deviation.

**FIGURE 1:**
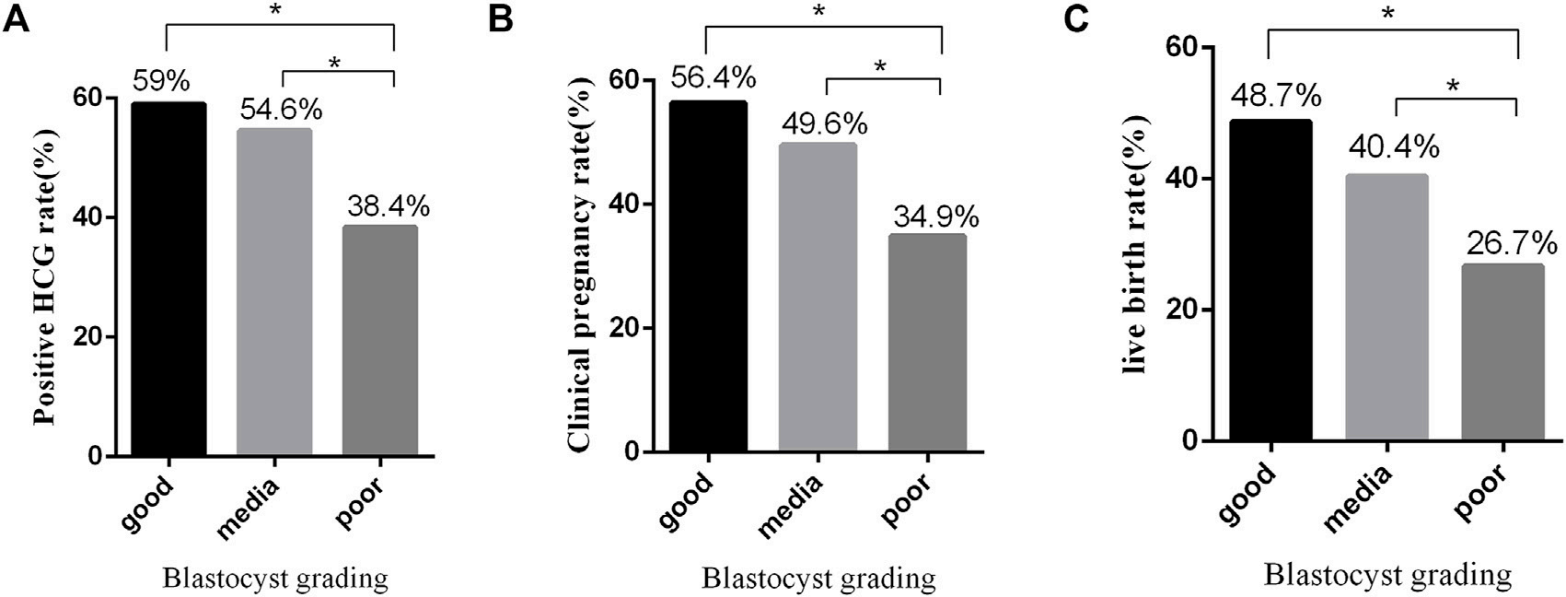
Effects of blastocyst grades on clinical outcomes of embryos. **(A)** Positive hCG rate from FET using embryos with different morphological grades. **(B)** Clinical pregnancy rate from FET using embryos with different morphological grades. **(C)** Live birth rate from FET using embryos with different morphological grades. *p ≤ 0.05 (chi-squared test).

We compared clinical embryo outcomes of transfer of single 1PN-derived blastocysts among the three groups. Transfer of the good-quality blastocysts achieved significant higher biochemical and clinical pregnancy ratios than those in the group of the poor-quality blastocysts (59% vs. 38.4%; *p* = 0.032 and 56.4% vs. 34.9%; *p* = 0.024, respectively, Figure 1A). Also, transfer of the medium-quality blastocysts achieved significant higher biochemical and clinical pregnancy ratios than those achieved in the poor-quality blastocyst group (54.6% vs. 38.4%; *p* = 0.018; 49.6% vs. 34.9%; *p* = 0.03, respectively, Figure 1B). However, there was no significant difference between the good-quality blastocysts and the medium-quality blastocysts in biochemical and clinical pregnancy ratios (59% vs. 54.6%; *p* = 0.627; 56.4% vs. 49.6%; *p* = 0.454). Furthermore, the live birth rates in groups of good, medium, and poor quality were 48.7%, 40.4% and 26.7%, respectively, exhibiting a blastocyst quality relative decrease (*p* = 0.353, *p* = 0.016, and *p* = 0.036, respectively, Figure 1C). Also, 1PN-derived blastocysts could be used for ET and cryopreservation since their use in FET cycles resulted in a high pregnancy and live birth rates. However, the clinical outcome significantly differed between various 1PN-derived blastocyst morphology groups. Our data indicated that the optimal clinical outcomes might result from the transferring of higher quality blastocysts (Table 3).

**TABLE 3:** Comparison of clinical outcomes of different morphological groups.

Morphological parameter	Positive hCG (%)	*P1*	OR	95% CI	*P2*	Clinical pregnancy rate (%)	*P1*	OR	95% CI	*P2*	Live birth (%)	*P1*	OR	95% CI	*P2*
Blastocyst															
Good	59.0% (23/39)	0.034	2.309	1.067–4.996	0.72	56.4% (22/39)	0.025	2.416	1.115–5.232	0.54	48.7% (19/39)	0.017	2.602	1.182–5.727	0.44
Medium	54.6% (77/141)	0.018	1.932	1.119–3.338	ref	49.6% (70/141)	0.031	1.840	1.059–3.199	ref	40.4% (57/141)	0.038	1.859	1.036–3.333	Ref
Poor	38.4% (33/86)	Ref				34.9% (30/86)	Ref				26.7% (23/86)	Ref			
ICM grade															
A	58.8% (20/34)	0.054	2.619	0.983–6.981	0.49	55.9% (19/34)	0.053	2.648	0.987–7.108	0.39	47.1% (16/34)	0.024	3.429	1.176–9.994	0.44
B	51.0% (101/198)	0.094	1.909	0.896–4.068	Ref	46.5% (92/198)	0.130	1.815	0.840–3.923	Ref	38.4% (76/198)	0.051	2.403	0.997–5.789	Ref
C	35.3% (12/34)	Ref				32.4% (11/34)	Ref				20.6% (7/34)	Ref			
TE grade															
A	52.9% (9/17)	0.307	1.761	0.594–5.220	0.81	52.9% (9/17)	0.202	2.036	0.684–6.063	0.90	41.2% (7/17)	0.337	1.729	0.565–5.291	0.88
B	53.2% (101/190)	0.059	1.776	0.979–3.223	Ref	48.4% (92/190)	0.086	1.699	0.928–3.108	Ref	39.5% (75/190)	0.140	1.611	0.855–3.038	Ref
C	39.0% (23/59)	Ref				35.6% (21/59)	Ref				28.8% (17/59)	Ref			
Expansion degree															
6	25% (4/16)	0.047	0.308	0.097–0.984	0.11	25% (4/16)	0.090	0.367	0.115–1.170	0.18	25% (4/16)	0.260	0.513	0.16–1.639	0.94
5	47.4% (9/19)	0.701	0.832	0.326–2.124	Ref	42.1% (8/19)	0.644	0.800	0.31–2.062	Ref	21.1% (4/19)	0.124	0.410	0.132–1.275	Ref
4 + 3	51.9% (120/231)	Ref	—	—	—	47.6% (110/231)	Ref	—	—	—	39.4% (91/231)	Ref	—	—	—

Note: OR, odds ratio; CI, confidence interval; TE, trophectoderm; ICM, inner cell mass; vs., *versus*; Ref, reference.

### Reference values of inner cell mass, trophectoderm development, and the expansion degree of blastocysts for clinical outcomes

Since TE, ICM, and the degree of blastocyst expansion are important in morphological grades, we next explored their reference values for embryo clinical outcomes. Although there was a tendency of decreases in biochemical and clinical pregnancy by ICM degrading, no statistical differences were observed (*p* > 0.05, Table 3). Only the live birth rate significantly decreased with the decline in the ICM grade (*p* < 0.05, Table 3). The group with A grade of ICM achieved a much higher live birth rate of 47.1%, while the group with C grade of ICM achieved a low live birth rate of 20.6% [*p* = 0.024, odds ratio (OR), 3.429; 95% CI, 1.176–9.994, Table 3]. It was also observed that the degree of blastocyst expansion was related with the biochemical rate but not the clinical pregnancy and live birth rate. Transferring of blastocysts with an expansion degree 6 yielded a lower biochemical rate than that of the blastocysts with an expansion degree 3–4 (25% vs. 51.9%; *p* = 0.047; odds ratio [OR], 0.308; 95% CI, 0.097–0.984, Table 3). However, the TE grade was found to have no effect on either the biochemical pregnancy rate, the clinical pregnancy rate, or the live birth rate (Table 3).

### Prediction of the live birth rate

To evaluate the prediction potential of blastocyst quality in the live birth rate, the blastocyst morphological parameters, ICM, TE, and expansion degree, were included in the multiple logistic regression analysis. As shown in Table 4, the prediction model provides the estimated probabilities of achieving a live birth depending on the composite quality score of the blastocysts. As examples for the model, a patient with transfer of a blastocyst with a score of 3-4AB, 5BB, or 6CC is predicted to have a probability of achieving a live birth of 52%, 25%, or 8%, respectively.

**TABLE 4:** Predicted live birth rate based on the multiple logistic regression model.

Expansion	ICM grade	TE grade	Live birth rate
3 + 4	A	A	45 (22–69)
3 + 4	A	B	52 (34–70)
3 + 4	A	C	37 (19–60)
3 + 4	B	A	37 (17–63)
3 + 4	B	B	44 (36–53)
3 + 4	B	C	30 (19–43)
3 + 4	C	A	20 (6–49)
3 + 4	C	B	25 (12–45)
3 + 4	C	C	15 (6–34)
5	A	A	26 (7–62)
5	A	B	32 (11–63)
5	A	C	20 (6–52)
5	B	A	20 (5–55)
5	B	B	25 (10–52)
5	B	C	15 (5–40)
5	C	A	10 (2–38)
5	C	B	13 (4–35)
5	C	C	7 (2–25)
6	A	A	28 (8–66)
6	A	B	35 (12–67)
6	A	C	22 (6–57)
6	B	A	22 (6–59)
6	B	B	28 (11–55)
6	B	C	17 (5–44)
6	C	A	11 (2–42)
6	C	B	14 (4–39)
6	C	C	8 (2–29)

TE, trophectoderm; ICM, inner cell mass.

## Discussion

Until now, 1PN-derived blastocyst transfer has been applied in multiple centers (Staessen et al., 1993; van der Heijden et al., 2009; Azevedo et al., 2014; Bradley et al., 2017; Capalbo et al., 2017; Si et al., 2019; Li et al., 2021), but the association between morphology parameters and clinical outcomes has not been elucidated clearly. To the best of our knowledge, this study was the first report to present the association of ICM, TE, and expansion degree grading with the clinical outcomes and the prediction value of these parameters for achieving live birth in 1PN-derived blastocyst transfer. Our data showed that the good-quality blastocysts obtained higher biochemical and clinical pregnancy and live birth, indicating that the conventional criteria of the morphological grading system (Gardner and Schoolcraft, 1999; Gardner et al., 2000) were also valuable for 1PN-derived blastocyst selection for transfer. Another strength of this study is that we provide the prediction model based on ICM grades, TE grades, and expansion degree, which simultaneously assess all developmental variables that can be used in embryo selection.

The correlation between blastocyst morphology and outcome of single 2PN-derived blastocyst transfers was evaluated in a large number of reports (Hill et al., 2013; Van den Abbeel et al., 2013; Rienzi et al., 2019) using the criteria of Gardner and Schoolcraft (Gardner et al., 2000). It was proved that the scores of ICM grades, TE grades, and expansion degree were significantly associated with pregnancies and live birth rates. Consistently, our data showed that the good-quality blastocysts achieved the highest pregnancy and live birth rate, and the medium blastocysts also owned better clinical outcomes than the poor blastocysts. Therefore, the overall grading of blastocyst quality based on the scores of ICM, TE, and expansion degree was still appropriate for 1PN-derived blastocyst transfer selection for higher pregnancy and live birth.

With respect to the association of morphological parameters with the clinical outcomes, there are many controversial studies. Goto et al. (2011) reported that the significant increases in the clinical pregnancy rate, viable pregnancy rate, and delivery rate were achieved with high-quality blastocyst transfer in 1,488 transfer cycles, while neither ICM nor TE affected the pregnancy outcome within the same blastocyst expansion. However, the extent of blastocyst expansion was found to be the most important parameter in another study (Van den Abbeel et al., 2013). It was also reported that an ICM containing many cells contributed to vital implantation (Richter et al., 2001; Kovacic et al., 2004) and may reduce the risk of pregnancy loss (Hill et al., 2013). However, the relationship of the morphological parameters with the clinical outcomes has not been clarified separately in those 1PN-derived blastocysts. In the current study, we found a significant association between the ICM grading and live birth. Our data showed that the live birth rate reached 47.1% in 1PN-derived blastocysts with ICM A, much higher than that of blastocysts with ICM C. Here, our data showed that the ICM grading, neither TE nor expansion degree, seemed the most important factor to influence live birth in 1PN-derived blastocyst transfer. Thus, the quality grade and ICM should be preferentially considered when transferring the vitrified-warmed blastocysts derived from monopronuclear zygotes. The same results but for 2PN-derived fresh blastocysts were presented by Kovacic et al. (2004).

Even though we did not find a correlation between TE grade and pregnancy potential and live births, its importance in euploid embryo competence has been reported due to its crucial role in the process of implantation in fresh or vitrified-warmed cycles (Norwitz et al., 2001; Ahlstrom et al., 2011). Also, the blastocyst expansion degree has been previously reported as predictive of implantation for euploid embryos (Van den Abbeel et al., 2013). Our data showed that they could not be assessed as a significant parameter for pregnancy or live birth after 1PN-derived blastocyst transfer. The TE and expansion degree were proposed as associated directly because a high-quality TE might involve a more efficient pumping of the ions, which in turn prompts the blastocyst expansion (Ahlstrom et al., 2011). However, both TE and expansion degree lost their significance in the analysis of the pure association with the clinical outcomes in our study, which might be resulted from the correction by the other morphological parameters. Nonetheless, all of these parameters were included in the prediction model of live birth, since the overall grading of blastocysts involving ICM, TE, and expansion degree showed a significant value for pregnancies and live births. Interestingly, the CC blastocysts, such as 3 + 4 CC, 5CC, and 6CC grade, still have 15%, 7%, and 8% live birth, respectively. It is important to recognize that despite little priority for these low-quality blastocysts, they could be a candidate for transfer, especially in those patients without competent embryos.

Our study is limited by the retrospective nature, and the embryo grading is inevitably subjected to intra- and inter-observer variations. The small number of groups of expansion degree 6 and 5 might weaken the conclusions regarding its association with clinical outcomes. Further prospective studies with larger sample sizes of transfer cycles to assess the prediction efficacy of our live birth model for transfer of 1PN-derived blastocysts are required.

Although there are many novel methods that are developed to select the high-competent embryos, such as genetic screening and time-lapse systems, embryo morphology evaluation under the microscope at different stages still remains valuable for the easiness, convenience, and invasiveness. In the current study, we present the valuable reference of traditional morphology grading in 1PN-derived blastocyst transfer. Our data supported that the overall quality grading was significantly associated with pregnancies and live births, and ICM seemed the most important factor in regard to live births. The strengths of the study are the performance of single blastocyst transfers and using live births as the measurement endpoint. The prediction model of live births will promote the selection of 1PN-derived blastocysts with high-developmental capacity. Although the clinical outcomes and live birth rates are high with good-quality 1PN-derived blastocysts, the blastocysts from normal fertilization (2PN) should be prioritized when selecting an embryo for transfer.

## Conclusion

1PN-derived blastocysts have a high potential to result in live births and should not be discarded. In 1PN transfer cycles, a higher overall blastocysts quality is shown to correlate most strongly with optimal pregnancy and live birth outcomes. The selection of high-quality blastocysts for transfer should consider the ICM score first. The prediction model of live births based on ICM, TE, and expansion degree may help predict successful pregnancy in 1PN single-blastocyst transfer cycles.

## Data Availability

The original contributions presented in the study are included in the article/Supplementary Material; further inquiries can be directed to the corresponding authors.
